# The fatty acid amide hydrolase inhibitor URB597 exerts anti-inflammatory effects in hippocampus of aged rats and restores an age-related deficit in long-term potentiation

**DOI:** 10.1186/1742-2094-9-79

**Published:** 2012-04-26

**Authors:** Niamh Murphy, Thelma R Cowley, Christoph W Blau, Colin N Dempsey, Janis Noonan, Aoife Gowran, Riffat Tanveer, Weredeselam M Olango, David P Finn, Veronica A Campbell, Marina A Lynch

**Affiliations:** 1 Department of Physiology, Trinity College, Trinity College Institute for Neuroscience, Dublin, 2, Ireland; 2 Pharmacology and Therapeutics, School of Medicine, NCBES Neuroscience Cluster and Centre for Pain Research, National University of Ireland, Galway, University Road, Galway, Ireland

**Keywords:** Age, Long-term potentiation (LTP), Endocannabinoids, Anandamide microglial activation, Hippocampus, Fatty acid amide hydrolase (FAAH)

## Abstract

**Background:**

Several factors contribute to the deterioration in synaptic plasticity which accompanies age and one of these is neuroinflammation. This is characterized by increased microglial activation associated with increased production of proinflammatory cytokines like interleukin-1β (IL-1β). In aged rats these neuroinflammatory changes are associated with a decreased ability of animals to sustain long-term potentiation (LTP) in the dentate gyrus. Importantly, treatment of aged rats with agents which possess anti-inflammatory properties to decrease microglial activation, improves LTP. It is known that endocannabinoids, such as anandamide (AEA), have anti-inflammatory properties and therefore have the potential to decrease the age-related microglial activation. However, endocannabinoids are extremely labile and are hydrolyzed quickly after production. Here we investigated the possibility that inhibiting the degradation of endocannabinoids with the fatty acid amide hydrolase (FAAH) inhibitor, URB597, could ameliorate age-related increases in microglial activation and the associated decrease in LTP.

**Methods:**

Young and aged rats received subcutaneous injections of the FAAH inhibitor URB597 every second day and controls which received subcutaneous injections of 30% DMSO-saline every second day for 28 days. Long-term potentiation was recorded on day 28 and the animals were sacrificed. Brain tissue was analyzed for markers of microglial activation by PCR and for levels of endocannabinoids by liquid chromatography coupled to tandem mass spectrometry.

**Results:**

The data indicate that expression of markers of microglial activation, MHCII, and CD68 mRNA, were increased in the hippocampus of aged, compared with young, rats and that these changes were associated with increased expression of the proinflammatory cytokines interleukin (IL)-1β and tumor necrosis factor-α (TNFα) which were attenuated by treatment with URB597. Coupled with these changes, we observed an age-related decrease in LTP in the dentate gyrus which was partially restored in URB597-treated aged rats. The data suggest that enhancement of levels of endocannabinoids in the brain by URB597 has beneficial effects on synaptic function, perhaps by modulating microglial activation.

## Introduction

Endocannabinoids are lipid-derived molecules which, in the brain, are synthesized by both astrocytes and microglia [1,2]. The two most studied endocannabinoids are anandamide (AEA) which is hydrolyzed by fatty acid amide hydrolase (FAAH) and 2-arachidonoylglycerol (2-AG), which is hydrolyzed by FAAH and monoacylglycerol lipase (MAGL) [3,4]. FAAH also catalyzes the hydrolysis of the *N*-acylethanolamines, *N*-palmitoyl ethanolamide (PEA), and *N*-oleoyl ethanolamide (OEA) [5], which, though not themselves endocannabinoids, can compete with AEA as substrates for FAAH and therefore increase AEA levels via the so-called ‘entourage effect’ [6]. To date, two cannabinoid receptors have been cloned; in the brain, CB_1_ receptors are expressed predominantly on neurons, whereas CB_2_ receptors are expressed mainly on immune cells, including microglia [7]. Constitutive expression of CB_1_ on neurons has been described, but expression of CB_2_ in the brain is low under resting conditions. However CB_2_ receptor expression on microglia increases markedly in conditions where neuroinflammatory changes occur for example in multiple sclerosis and Alzheimer’s disease [8,9] and in the lesioned striatum in an animal model of Huntington’s disease [10]. Interestingly, increased CB_2_ receptor expression has been demonstrated on the microglia that surround amyloid-β (Aβ)-containing plaques in Alzheimer’s disease [8,11].

The neuroprotective effects of endocannabinoids have been carefully described by several groups, for example following neurotoxic stimuli [12,13] and Aβ treatment [14]. The ability of cannabinoids to modulate the adaptive and innate branches of the immune system has been recognized for several years [15] and, in the context of the CNS, a great deal of emphasis has been placed on evaluating the effects of cannabinoids in multiple sclerosis and particularly the animal model, experimental autoimmune encephalomyelitis (EAE; [16]). The ability of the cannabinoid delta(9)-tetrahydrocannibinol (THC) to decrease inflammation in the spinal cord of animals in which EAE was induced, was reported over two decades ago [17] and several studies have supported this finding with recent evidence indicating that symptoms and inflammatory changes, including microglial activation, were more profound in CB_2_ receptor knockout mice [18]. The cannabinoid agonist, (R)-(+)-[2,3-dihydro-5-methyl-3-(4-morpholinylmethyl)-pyrrolo[1,2,3-de-1,4benzoxazin-6-yl]-1-naphthalenyl-methanone mesylate (WIN-55,212-2) has been shown to attenuate the microglial activation observed in brain of animals which received an intracerebraventricular injection of Aβ_25-35_ and it also attenuated the Aβ-associated decrease in neuronal proteins and deficits in spatial learning [11]. Consistently, a number of *in vitro* studies have demonstrated that endocannabinboids and/or synthetic cannabinoids attenuate microglial activation induced by interferon-γ (IFNγ) [19], Aβ [11], or lipopolysaccharide (LPS) [20,21].

A good deal of evidence indicates that microglial activation increases with age and this is closely linked with the age-related deficit in synaptic plasticity, particularly long-term potentiation (LTP) [22,23] and it has been shown that LTP is sustained in aged rats by interventions which decrease microglial activation [22,24,25]. An age-related deficit in spatial learning, which is another form of synaptic plasticity, has also been reported and interestingly, when aged rats were treated with WIN-55,212-2, performance in a spatial learning task improved and this was correlated with a decrease in the number of activated microglia in CA3 but not in the dentate gyrus [26].

We hypothesized that administration of the FAAH inhibitor, URB597, which, by decreasing AEA hydrolysis, would increase endocannabinoid tone and therefore decrease the age-related microglial activation and consequently enable aged rats to sustain LTP. The data indicate that administration of URB597, increased brain tissue concentrations of AEA, and other *N-acylethanolamines*, attenuated the increased expression of several markers of microglial activation in aged animals and improved the ability of aged rats to sustain LTP.

## Materials and methods

### Animals

Young (3 months; 250–350 g) and aged (26–30 months; 550–600 g) male Wistar rats (B&K Universal, Hull, UK) were housed in a controlled environment (temperature 20-22°C; 12:12 h light/dark cycle) in the BioResources Unit, Trinity College, Dublin. Animals had free access to food and water and were maintained under veterinary supervision for the duration of the experiment. Young and aged rats were randomly divided into those which received subcutaneous injections of the FAAH inhibitor URB597 (1 mg/kg; dissolved in 30% DMSO, saline) every second day and controls which received subcutaneous injections of 30% DMSO-saline every second day for 28 days. All experiments were carried out under license from the Department of Health and Children (Ireland) and with ethical approval from the Trinity College Ethical Committee.

### Analysis of LTP *in vivo*

Rats were anaesthetized by intraperitoneal injection of urethane (1.5 g/kg) and the absence of a pedal reflex was considered to be an indicator of deep anesthesia; in some animals a top-up dose of urethane (to a maximum of 2.0 g/kg) was required to establish deep anesthesia. The ability of rats to sustain LTP in perforant path-granule cell synapses in response to tetanic stimulation of the perforant path was assessed as previously described [27]. Briefly, a bipolar stimulating electrode was stereotaxically positioned in the perforant path (4.4 mm lateral to Lambda) and a unipolar recording electrode was placed in the dorsal cell body region of the dentate gyrus (2.5 mm lateral and 3.9 mm posterior to Bregma). Following a period of stabilization, test shocks were delivered at 30-s intervals and responses were recorded for 10 min to establish stable baseline recordings. LTP was induced by delivering three trains of high-frequency stimuli (250 Hz for 200 ms; 30 second inter-train interval). Recording at test shock frequency resumed for the remainder of the experiment. The slope of the excitatory post-synaptic potential (EPSP) was used as a measure of excitatory synaptic transmission in the dentate gyrus.

At the end of the experiment, rats were killed by cervical dislocation and brain tissue was dissected free. Tissue was snap-frozen and used to prepare mRNA for PCR analysis or for the quantification of endocannbinoids.

### Real-time PCR analysis of cytokines and cell surface markers

Total RNA was extracted from snap-frozen hippocampal and cortical tissue using a NucleoSpin® RNAII isolation kit (Macherey-Nagel Inc., Germany) according to the manufacturer’s instructions. RNA integrity and total RNA concentration were assessed, and cDNA synthesis was performed as described previously [28]. Real-time PCR was performed using Taqman Gene Expression Assays (Applied Biosystems, Germany) which contain forward and reverse primers, and a FAM-labeled MGB Taqman probe for each gene of interest. The assay IDs for the genes examined in this study were as follows: *MHCII* (Rn01768597_m1), *CD40* (Mm00441895_m1), *CD11b* (Mm001271265_m1), *CD68* (Rn01495631_g1), *IL-1β* (Rn00580432_m1), TNFα (Mm00443258_m1), and *IL-6* (Mm00446191_m1). All real-time PCR was conducted using an ABI Prism 7300 instrument (Applied Biosystems, Germany). A 20 μl volume was added to each well containing 8 μl of cDNA (1:4 dilution), 1 μl of target gene primer, and 10 μl of Taqman® Universal PCR Master Mix). Samples were assayed in duplicate in one run (40 cycles), which consisted of three stages, 95°C for 10 min, 95°C for 15 s for each cycle (denaturation), and finally the transcription step at 60°C for 1 min. β-actin was used as endogenous control to normalize gene expression data, and β-actin expression was conducted using a gene expression assay containing forward and reverse primers (primer limited) and a VIC-labeled MGB Taqman probe from Applied Biosystems (Germany; Assay ID: 4352341E). Gene expression was calculated relative to the endogenous control samples and to the control sample giving an RQ value (2− DDCt, where CT is the threshold cycle).

### Quantitation of endocannabinoids and N-acylethanolamines in cerebellar tissue using liquid chromatography coupled to tandem mass spectrometry (LC-MS/MS)

Brains from the young and aged, vehicle or URB597 treated rats were removed rapidly and the cerebellum was gross-dissected (average weight of tissue samples = 158.26 mg), snap-frozen on dry ice and stored at -80^0^ C prior to extraction and determination of the concentrations of the endocannabinoids anandamide (AEA) and 2-arachidonoyl glycerol (2-AG) and the related *N*-acylethanolamines *N*-palmitoyl ethanolamide (PEA) and *N*-oleoyl ethanolamide (OEA) by liquid chromatography coupled to tandem mass spectrometry (LC-MS/MS) as described previously [29,30]. Each tissue sample was first homogenized in 400 μL 100% acetonitrile containing known fixed amounts of deuterated internal standards (0.014 nmol AEA-d8, 0.48 nmol 2-AG-d8, 0.016 nmol PEA-d4, 0.015 nmol OEA-d2). Homogenates were centrifuged at 14,000 g for 15 min at 4°C and the supernatant was collected and evaporated to dryness in a centrifugal evaporator. Lyophilized samples were re-suspended in 40 μL 65% acetonitrile and 2 μL were injected onto a Zorbax® C18 column (150 × 0.5 mm internal diameter) from a cooled autosampler maintained at 4°C (Agilent Technologies Ltd, Cork, Ireland). Mobile phases consisted of A (HPLC grade water with 0.1% formic acid) and B (acetonitrile with 0.1% formic acid), with a flow rate of 12 μL/min. Reversed-phase gradient elution began initially at 65% B and over 10 min was ramped linearly up to 100% B. At 10 min, the gradient was held at 100% B up to 20 min. At 20.1 min, the gradient returned to initial conditions for a further 10 min to re-equilibrate the column. The total run time was 30 min. Under these conditions, AEA, 2-AG, PEA, and OEA eluted at the following retention times: 11.36 min, 12.8 min, 14.48 min, and 15.21 min, respectively. Analyte detection was carried out in electrospray-positive ionization mode on an Agilent 1100 HPLC system coupled to a triple quadrupole 6460 mass spectrometer (Agilent Technologies Ltd., Cork, Ireland). Instrument conditions and source parameters including fragmentor voltage and collision energy were optimized for each analyte of interest prior to assay of samples. Quantitation of target endocannabinoids was achieved by positive ion electrospray ionization and multiple reactions monitoring (MRM) mode, allowing simultaneous detection of the protonated precursor and product molecular ions [M + H+] of the analytes of interest and the deuterated forms of the internal standards. Quantitation of each analyte was performed by determining the peak area response of each target analyte against its corresponding deuterated internal standard. This ratiometric analysis was performed using Masshunter Quantitative Analysis Software (Agilent Technologies Ltd., Cork, Ireland). The amount of analyte in unknown samples was calculated from the analyte/internal standard peak area response ratio using an 11-point calibration curve constructed from a range of concentrations of the non-deuterated form of each analyte and a fixed amount of deuterated internal standard. The values obtained from the Masshunter Quantitative Analysis Software are initially expressed in ng per mg of tissue by dividing by the weight of the punched tissue. To express values as nmol or pmol per mg the corresponding values are then divided by the molar mass of each analyte expressed as ng/nmole or pg/pmole. Linearity (regression analysis determined R^2^ values of 0.99 or greater for each analyte) was determined over a range of 75 ng to 71.5 fg except for 2-AG which was 750 ng to 715 fg. The limit of quantification was 1.32 pmol/g, 12.1 pmol/g, 1.5 pmol/g, and 1.41 pmol/g for AEA, 2-AG, PEA, and OEA, respectively.

### Statistical analysis

Prism GraphPad® was used for statistical analysis. Data were analyzed using analysis of variance (ANOVA) with Newman Keuls post-hoc test to determine which conditions were significantly different from each other. Data are expressed as means with standard errors.

## Results

**Figure 1:**
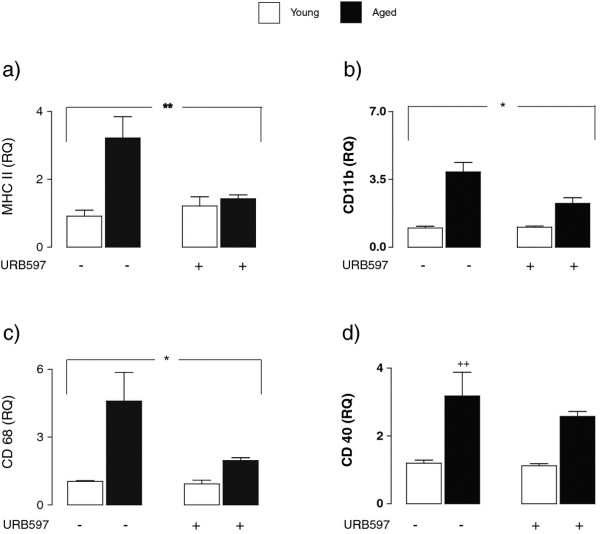
**Age-related microglial activation in hippocampus is attenuated by URB597.** URB597 attenuated the age-related increase in MHCII mRNA, CD68 mRNA, and CD11b mRNA in hippocampal tissue and the analysis revealed a significant age x treatment interaction for MHCII mRNA (F_(1,22)_ = 8.84, **P < 0.01; 2-way ANOVA; **a**), CD11b mRNA (F_(1,22)_ = 6.22, *P < 0.05; **b**), and CD68 mRNA (F_(1,22)_ = 4.80, *P < 0.05; **c**). A significant age effect was observed in the case of CD40 mRNA (F_(1,21)_ = 14.09, ^++^*P* < 0.01; **d**).

MHCII, CD68, and CD11b mRNA were increased in hippocampal tissue prepared from aged, compared with young, rats and the evidence indicates that these measures of microglial activation were decreased in tissue prepared from aged rats which were treated with URB597. A significant age x treatment interaction was observed for MHCII mRNA (F_(1,22)_ = 8.84, **P < 0.01; Figure 1a), CD11b mRNA (F_(1,22)_ = 6.22, *P < 0.05; Figure 1b) and CD68 mRNA (F_(1,22)_ = 4.80, *P < 0.05; Figure 1c), whereas a significant age effect was observed in the case of CD40 mRNA (F_(1,21)_ = 14.09, ^++^*P* < 0.01; Figure 1d).

**Figure 2:**
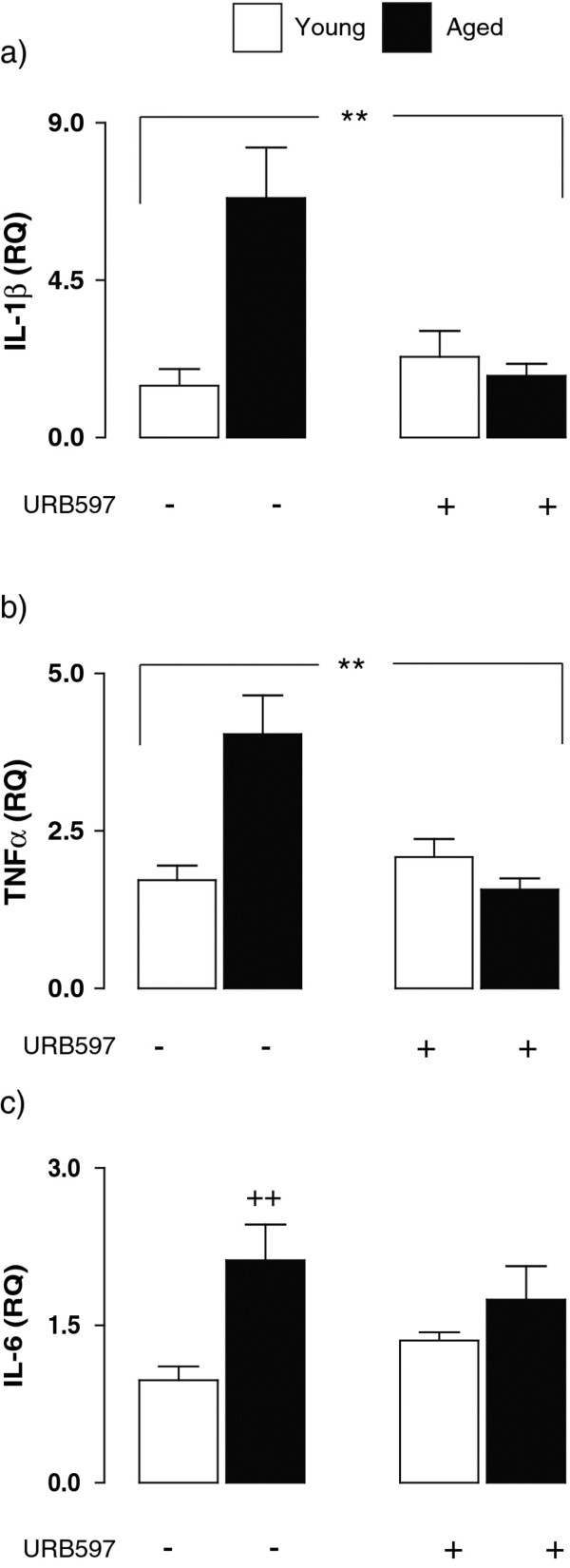
**URB597 treatment attenuates the age-related increase in inflammatory cytokines in hippocampus.** IL-1β, TNFα, and IL-6 mRNA were increased in hippocampal tissue prepared from aged, compared with young, rats; a significant age x treatment effect was observed for IL-1β and TNFα (F_(1,22)_ = 5.096, **P < 0.01; and F_(1,21)_ = 16.16, **P < 0.01; 2-way ANOVA; **a** and **b**, respectively) and a significant age effect was observed in the case of IL-6 (F_(1,21)_ = 29.98, ^++^*P* < 0.001; **c**).

Activated microglia are a major source of inflammatory cytokines and, here, we assessed whether the age-related increase in microglial activation was associated with evidence of increased production of inflammatory cytokines. There was an increase in IL-1β, TNFα, and IL-6 mRNA in hippocampal tissue prepared from aged, compared with young, rats; a significant age x treatment effect was observed in both IL-1β and TNFα (F_(1,22)_ = 5.096, **P < 0.01; 2-way ANOVA; Figure 2a and F_(1,21)_ = 16.16, **P < 0.01; Figure 2b, respectively) whereas a significant age effect was observed in the case of IL-6 (F_(1,21)_ = 29.98, ^++^*P* < 0.01; 2-way ANOVA; Figure 2c).

**Figure 3:**
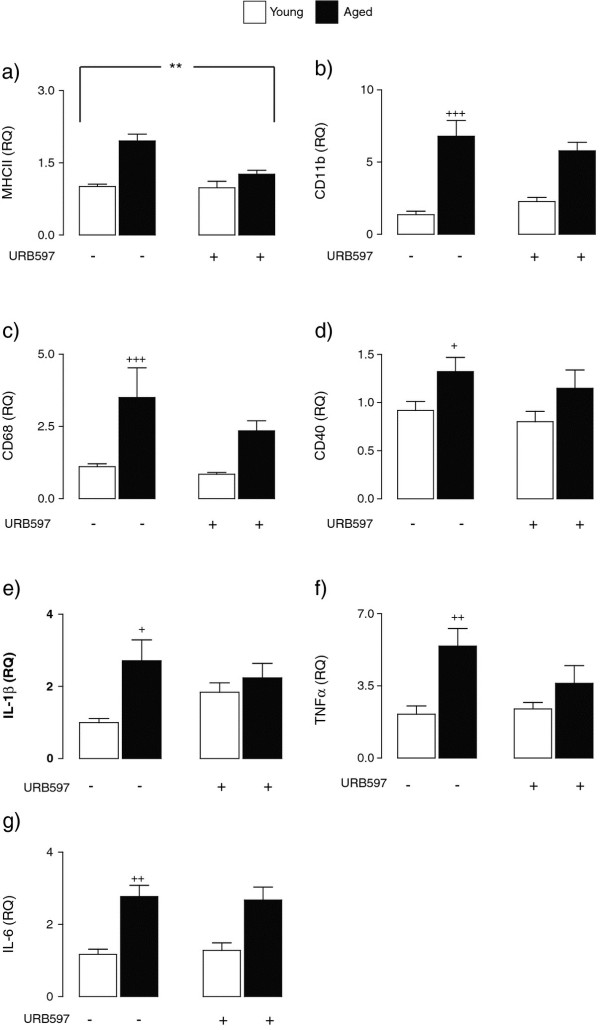
**URB597 treatment attenuates age-related changes in cortex.** Increased expression of MHCII, CD11b, CD68, and CD40 mRNA were observed in cortex of aged, compared with young, rats. A significant age x treatment effect in the case of MHCII mRNA (F_(1,23)_ = 9.11, **P < 0.01; 2-way ANOVA; **a**) and significant age effects were observed in the case of CD11b (F_(1,20)_ = 44.86, ^+++^*P* < 0.001; **b**), CD68 (F_(1,22)_ = 14.81, ^+++^*P* < 0.001; **c**) and CD40 mRNA (F_(1,20)_ = 5.62, ^+^*P* < 0.05; **d**). Significant age effects were also observed in IL-1β (F_(1,22)_ = 5.97, ^+^*P* < 0.05; 2-way ANOVA; **e**), TNFα (F_(1,23)_ = 9.98, ++*P* < 0.01; **f**) and IL-6 (F_(1,26)_ = 20.91, ^++^*P* < 0.01; **g**).

Similar age-related increases in expression of MHCII, CD68, CD40, and CD11b mRNA were observed in cortical tissue. Analysis of the data indicated a significant age x treatment interaction in the case of MHCII mRNA (F_(1,23)_ = 9.11, **P < 0.01; 2-way ANOVA; Figure 3a) whereas significant age effects were observed in the case of CD11b (F_(1,20)_ = 44.86, ^+++^*P* < 0.001; Figure 3b), CD68 (F_(1,22)_ = 14.81, ^+++^*P* < 0.001; Figure 3c), and CD40 mRNA (F_(1,20)_ = 5.62, ^+^*P* < 0.05; Figure 3d). In parallel with the changes in hippocampus, expression of IL-1β, TNFα, and IL-6 mRNA were increased in cortical tissue prepared from aged, compared with young, rats. A significant age effect was observed in the case of IL-1β (F_(1,22)_ = 5.97, ^+^*P* < 0.05; 2-way ANOVA; Figure 3e), TNFα (F_(1,23)_ = 9.98, ^++^*P* < 0.01; Figure 3f) and IL-6 (F_(1,26)_ = 20.91, ^++^*P* < 0.01; Figure 3g). URB597 had no significant effects on the age related increases in the expression of IL-1β, TNFα, or IL-6 mRNA in the cortex (Figure 3e, f, and g).

**Figure 4:**
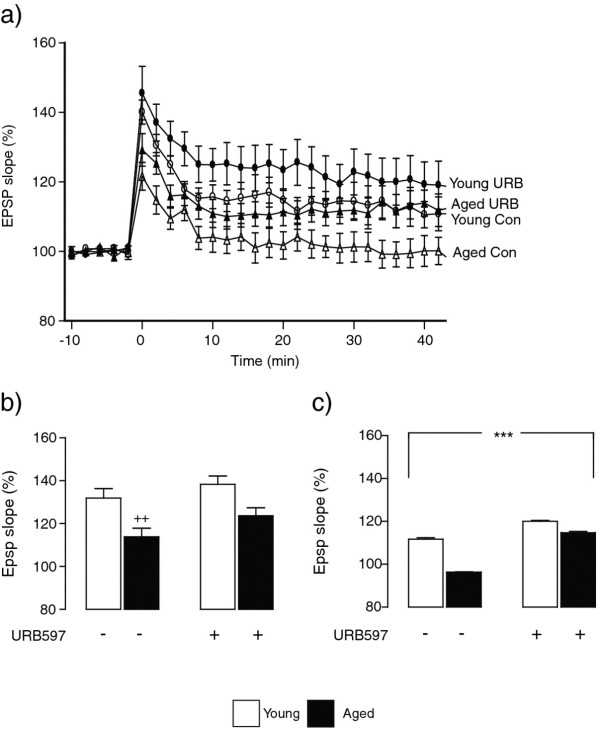
**URB597 treatment attenuates the age-related decrease in LTP in dentate gyrus.** (**a**) Delivery of a high frequency train of stimuli to the perforant path at time 0 induced an immediate and sustained increase in EPSP slope in control young rats and this effect was markedly decreased in control aged rats. Aged rats treated with URB597 sustained LTP in a manner similar to young animals. (**b**) The mean changes in EPSP slope in the 5 min immediately following tetanic stimulation revealed a significant age effect (F_(1,8)_ = 16.73, ++*P* < 0.01; 2-way ANOVA; b) and analysis of the data in the last 5 min of recording indicated that there was a significant age x treatment interaction (F_(1,8)_ = 444.1, ***P < 0.001; 2-way ANOVA; c).

A key question was to assess whether URB597, by modulating microglial activation, might improve the ability of aged rats to sustain LTP. Delivery of a high frequency train of stimuli to the perforant path induced an immediate and sustained increase in EPSP slope in young control-treated rats (Figure 4a) whereas the initial increase in EPSP slope in aged control-treated rats was temporary and the mean value returned to baseline after about 10 min. However aged rats which received URB597 sustained LTP to the same extent as young control-treated rats and URB597 enhanced the ability of young rats to sustain LTP. Analysis of the mean data in the 5 min immediately following tetanic stimulation revealed a significant age effect (F_(1,8)_ = 16.73, ^++^*P* < 0.01; 2-way ANOVA; Figure 4b) while analysis of the data in the last 5 min of recording indicated that there was a significant age x treatment interaction (F_(1,8)_ = 444.1, ***P < 0.001; 2-way ANOVA; Figure 4c) indicating that treatment of aged animals with URB597 attenuated the impairment in LTP.

**Figure 5:**
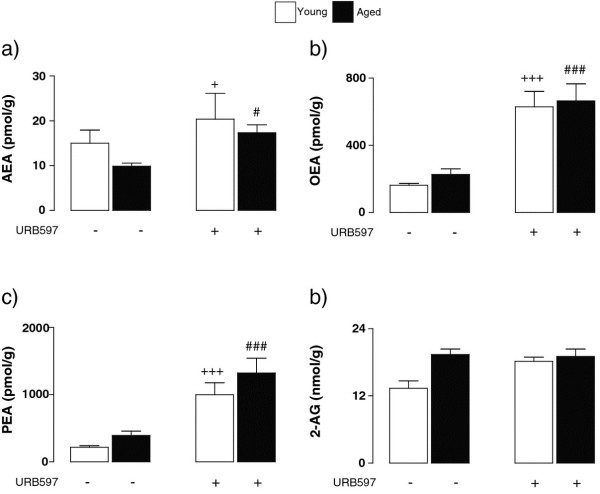
**URB597 treatment increased concentrations of the endocannabinoid AEA and the*N*-acylethanolamines OEA and PEA.** Analysis of the analyte concentrations revealed a significant treatment effect for AEA (F_(1,23)_ = 6.29, +*P* < 0.05; #*P* < 0.05; 2-way ANOVA; **a**), OEA (F_(1,23)_ = 35.30, ^+++^*P* < 0.001; ^###^*P* < 0.001; **b**) and PEA (F_(1,23)_ = 29.21, ^+++^*P* < 0.001; ^###^*P* < 0.001; **c**) and a significant age x treatment interaction was observed in the case of 2-AG (F_(1,23)_ = 4.86, **P < 0.01; **d**).

Tissue concentrations of endocannabinoids and related *N*-acylethanolamines in the cerebellum were assessed by liquid chromatography coupled to tandem mass spectrometry and analysis of the data obtained for AEA revealed a significant treatment effect (F_(1,23)_ = 6.29, ^+^*P* < 0.05; ^#^*P* < 0.05; 2-way ANOVA; Figure 5a). Similarly, analysis of the data obtained for OEA and PEA indicated that there were significant treatment effects in both cases (F_(1,23)_ = 35.30, ^+++^*P* < 0.001; ^###^*P* < 0.001; 2-way ANOVA; Figure 5b and c). No significant treatment effect was observed in the case of 2-AG (Figure 5d).

## Discussion

The aim of this study was to assess whether the age-related increase in microglial activation and the associated decrease in LTP were attenuated by chronic administration of the FAAH inhibitor, URB597. The data show that URB597 increased AEA, OEA, and PEA and that this was accompanied by a URB597-associated attenuation of the age-related neuroinflammatory changes and also the age-related impairment in LTP.

Increased expression of MHCII, CD11b, CD68, and CD40, which are commonly-used markers of microglial activation, were observed in hippocampus and also the cortex of aged, compared with young rats. This concurs with previously-reported findings which demonstrated that these, and other markers of microglial activation, were increased with age [24,31,32]. One of the most significant findings of this study is that these changes were attenuated in hippocampal and cortical tissue prepared from aged rats which received URB597. Cannabinboids are known to modulate certain aspects of microglial function *in vitro*; for instance the phytocannabinoid THC and the non-hydrolyzable analogue of anandamide, methanandamide, decreased LPS-induced cytokine production from rat cortical glial cells [21,33], while AEA and 2-AG, as well as a number of synthetic cannabinoids, inhibited the LPS-induced release of TNFα [20] and the generation of nitrites [34] from cultured glial cells. At least in some studies [20,33], the actions of the cannabinoids were not CB receptor mediated. There are other reports of a similar modulatory effect of synthetic cannabinoids on microglial activation *in vitro* including their ability to attenuate the ATP-induced increase in intracellular calcium concentration [34] and the neurotoxicity induced by Aβ-treated microglia [11]. Similarly the LPS-induced release of TNFα and IL-1β from cultured astrocytes was attenuated by both anandamide and the anandamide uptake inhibitor, UCM707 [35]. In addition to these effects *in vitro*, it has been shown that the increase in microglial activation induced by the central administration of LPS to rats for 21 days [26] or by daily intracerebroventricular injection of Aβ_25-35_ for 7 days [11] was attenuated by subcutaneously- or centrally-administered WIN55,212-2, respectively.

While a number of cells produce inflammatory cytokines, activated microglia are considered to be a primary source of cytokines such as IL-1β, IL-6, and TNFα in the brain. The present data indicate that the age-related increase in markers of microglial activation are accompanied by an increase in these cytokines confirming earlier reports of a similar parallel [36]. The increase in cytokine production was markedly reduced in hippocampal tissue prepared from aged rats which received URB597 providing evidence of an anti-inflammatory effect of the FAAH inhibitor. URB597 treatment has been shown to decrease LPS-induced PGE2 production in cultured microglia though it did not attenuate the increases in COX2 and iNOS [37]. Intra-peritoneal injections of URB597 have also been shown to reduce LPS induced increases in IL-1β in the hypothalamus in Sprague-Dawley rats [38]. The synthetic cannabinoid, dexanabinol, which facilitated recovery and decreased cell death, reduced hippocampal expression of TNFα and IL-1β in the hippocampus after traumatic brain injury [39]. Perhaps in contrast with this is the report that the CB_2_ agonist JWH-133, which decreased infarct volume following middle cerebral artery occlusion, did not attenuate the increase in TNF or IL-1β in ischaemic brain tissue [40].

In the past few years, it has become increasingly clear that neuroinflammation negatively impacts on neuronal plasticity [36] and specifically that LTP is decreased when microglial activation and/or inflammatory cytokine production is increased in hippocampus [24,36,41]. The present findings provide support for this inverse correlation, specifically demonstrating that LTP was decreased in dentate gyrus of aged rats. Significantly, the age-related deficit in LTP was attenuated by treatment with URB597 in parallel with its ability to decrease the expression of several markers of microglial activation and the production of inflammatory cytokines in the hippocampus. These changes concur with the findings of previous studies which indicated that when the age-related increase in microglial activation is attenuated, for example with minocycline [25], the anti-inflammatory cytokine IL-4 [23], the polyunsaturated fatty acids EPA and DPA [32], the cholesterol-lowering HMGCoA reductase, atorvastatin [41], and the PPARγ activator, rosiglitazone [42], then the ability of aged rats to sustain LTP is improved.

A facilitatory effect of cannabinoids on other forms of synaptic plasticity has also been reported. Thus the synthetic cannabinoid, WIN55,212-2, attenuates the impaired spatial learning observed in rats which received Aβ_25-35_ intracerebroventricularly for 7 days [11]. This effect was coupled with changes in neuronal markers calbindin and α-tubulin in tissue prepared from the frontal cortex of mice. Similarly intraperitoneal administration of WIN55,212-2 or cannabidiol for 3 weeks attenuated the cognitive impairment induced by a single injection of Aβ, although the CB_2_ agonists, 1,1-dimethylbutyl-1-deoxy-Δ^9^-tetrahydrocannabinol [JWH-133] and 4-[4-(1,1-dimethylheptyl)-2,6-dimethoxyphenyl]-6,6-dimethyl-bicyclo[3.1.1]hept-2-ene-2-methanol [HU-308] did not [34]; in this case the Aβ-induced increase in IL-6 was attenuated by WIN55,212-2 or cannabidiol prompting the authors to conclude that the effect of the cannabinoids resulted from modulation of glial activation. The correlation between glial activation and spatial learning is not absolute since it has been reported that while the increase in microglial activation induced by the central administration of LPS for 21 days was attenuated by WIN55,212-2, treatment with WIN55,212-2 exacerbated the deficit in spatial learning [26]. However the same group reported that when aged rats were treated with WIN-55,212-2, performance in a spatial learning task improved and this was correlated with a decrease in the number of activated microglia in CA3 but not dentate gyrus [26].

The effect of URB597 in the present study can be attributed to its ability to increase the concentration of endocannabinoids in the brain. The data indicate that the 28-day URB597 treatment regime used here increases concentrations of AEA, as well as two other fatty acid ethanolamides, PEA and OEA in the brain. The anti-inflammatory effects of AEA have been well documented both *in vitro* and *in vivo*[20,43-46] and both PEA and OEA possess anti-inflammatory properties [47,48]. While PEA appears to lack CB_1_ receptor binding activity, it interacts with the CB_2_ receptor which probably mediates its analgesic and anti-inflammatory effects [48-50]. In contrast, OEA may not interact with either CB_1_ or CB_2_ receptors, but rather engage one of the recently-described G protein-coupled orphan receptors [51]. It is possible that any of these endocannabinoids/*N*-acylethanolamines, that are increased following URB597 treatment, may contribute to the anti-inflammatory effects described in the present study.

One of the challenges in neuroscience is to identify the age-related changes in the brain which present the most significant risks for development of neurodegenerative diseases and to reduce these changes. In addition to the findings presented here, a good deal of evidence suggests that neuroinflammation, probably functionally linked with microglial activation, is one such change. We demonstrate that increasing endocannabinoid tone provides a mechanism by which the age-related microglial activation and deficit in synaptic plasticity can be attenuated.

## Competing interests

The authors declare that they have no competing interests.

## Authors’ contributions

NM contributed to design of the study, performed the majority of Real-Time PCR experiments, analyzed, reviewed and organized the data; TRC performed all LTP-related experiments; CND performed some of the Real-Time PCR experiments; CWB, JN, AG, and RT were involved in the URB597 treatment regime of the animals; WMO and DPF performed the mass-spectrometry experiments and VAC and MAL directed the overall study, wrote and reviewed the manuscript. All authors have read, reviewed, and approved the final manuscript.
